# Comparison of ^18^F-based PSMA radiotracers with [^68^Ga]Ga-PSMA-11 in PET/CT imaging of prostate cancer—a systematic review and meta-analysis

**DOI:** 10.1038/s41391-023-00755-2

**Published:** 2023-11-28

**Authors:** Siyu Huang, Sean Ong, Dean McKenzie, Adam Mirabelli, David C. Chen, Thilakavathi Chengodu, Declan G. Murphy, Michael S. Hofman, Nathan Lawrentschuk, Marlon Perera

**Affiliations:** 1 Department of Surgery, University of Melbourne, Parkville, VIC Australia; 2 EJ Whitten Prostate Cancer Research Centre, Epworth HealthCare, Melbourne, VIC Australia; 3 Research Development & Governance Unit, Epworth HealthCare, Melbourne, VIC Australia; 4 Department of Health Science and Biostatistics, Swinburne University of Technology, Melbourne, VIC Australia; 5 Prostate Cancer Theranostics and Imaging Centre of Excellence, Molecular Imaging and Therapeutic Nuclear Medicine, Peter MacCallum Cancer Centre, Melbourne, VIC Australia; 6 Sir Peter MacCallum Department of Oncology, University of Melbourne, Melbourne, VIC Australia; 7 Young Urology Researchers Organisation (YURO), Melbourne, VIC Australia; 8 Division of Cancer Surgery, Peter MacCallum Cancer Centre, Melbourne, VIC Australia; 9 Department of Urology, Royal Melbourne Hospital, Parkville, VIC Australia; 10 Department of Surgery, Austin Health, The University of Melbourne, Melbourne, VIC Australia

**Keywords:** Prostate cancer, Outcomes research

## Abstract

**Background:**

Prostate-specific membrane antigen (PSMA) positron emission tomography (PET)/computed tomography (CT) has become an increasingly established imaging modality in the staging of prostate cancer (PCa). Numerous PSMA-based tracers are currently available, however, there is a lack of consensus on the optimal radiotracer(s) for PSMA PET/CT. This study aims to investigate whether Fluorine-18 (^18^F)-labelled PSMA PET/CT is significantly different from Gallium-68 (^68^Ga) in primary diagnosis and/or secondary staging of prostate cancer following biochemical recurrence.

**Methods:**

A critical review of MEDLINE, EMBASE, PubMed and Web of Science databases was performed in May 2023 according to the Preferred Reporting Items for Systematic Review and Meta-analysis (PRISMA) statement. Studies that directly compared ^18^F-based PSMA radiotracers and [^68^Ga]Ga-PSMA-11 in terms of the normal organ SUV or the lesion SUV or the detection rate were assessed. Quality was assessed using the Quality Assessment of Diagnostic Accuracy Studies-2 (QUADAS-2).

**Results:**

Twenty-four studies were analysed. [^18^F]DCFPyL and [^18^F]PSMA-1007 were the two most commonly studied ^18^F based PSMA tracers. [^18^F]JK-PSMA-7, [^18^F]rhPSMA-7, [^18^F]AlF-PSMA-11 were the new tracers evaluated in a limited number of studies. Overall, [^18^F]DCFPyL was observed to have a similar lesion detection rate to [^68^Ga]Ga-PSMA-11 with no increase in false positive rates. [^18^F]PSMA-1007 was found to have a greater local lesion detection rate because of its predominant hepatobiliary excretory route. However, [^68^Ga]Ga-PSMA-11 was observed to have a similar local lesion detection rate in studies that administer patients with furosemide prior to the scan. In addition, [^18^F]PSMA-1007 was found to have a significant number of benign bone uptakes.

**Conclusions:**

[^18^F]DCFPyL was observed to be similar to [^68^Ga]Ga-PSMA-11. [^18^F]PSMA-1007 was observed to be less preferrable to [^68^Ga]Ga-PSMA-11 due to its high benign bone uptakes. Overall, there was not enough evidence in differentiating the radiotracers based on their clinical impacts.

## Introduction

Prostate cancer (PCa) is the second most common cancer in men worldwide [1]. Management of primary and recurrent PCa relies on accurate staging of the cancer through imaging and biopsies. As an emerging imaging modality, prostate surface membrane antigen (PSMA) positron emission tomography-computed tomography (PET/CT) has been approved by the United States Food and Drug Administration (FDA) and is recommended for detecting metastases and restaging PCa in cases of biochemical recurrence (BCR) [2]. The ProPSMA study has also proven that PSMA PET/CT provides superior accuracy to the combined findings of CT and bone scanning in primary staging of PCa [3]. Though magnetic resonance imaging remains the standard for detection of local lesions within the prostate gland, there is growing evidence to support the role of PSMA PET/CT for the same task [4].

PSMA is a transmembrane glycoprotein involved in the enzymatic process of glutamate release [5]. Contrary to its name, it is physiologically expressed in many organs and tissues, including the lacrimal and salivary glands, the kidney, the liver and the gastrointestinal tract. Furthermore, its expression can also be seen in the neovasculature of several solid, non-prostatic tumours [6]. In PCa, >90% of cells express PSMA, with higher rates of expressions in higher-grade cancer [7].

FDA has approved the use of one ^68^Ga-based radiotracer, [^68^Ga]Ga-PSMA-11 (also known as [^68^Ga]Glu-Urea-Lys(Ahx)-HBED-CC) in 2020 [2] and two ^18^F-based radiotracers, [^18^F]DCFPyL and [^18^F]rhPSMA-7 in 2021 and 2023 respectively [8, 9] in PSMA PET/CT. [^68^Ga]Ga-PSMA-11 is the most extensively studied and most widely used radiotracer. On the other hand, there is a paucity of experience of data with [^18^F]rhPSMA-7—whether it has benign bone uptake is not fully established. Over the years, more ^18^F-based PSMA radiotracers have been synthesised and assessed against [^68^Ga]Ga-PSMA-11 in terms of the diagnostic performance and the cost of production, including [^18^F]PSMA-1007, [^18^F]AIF-PSMA-11, and [^18^F]JK-PSMA-7. ^18^F is theoretically able to offer a higher image resolution owing to its lower end-point positron energy and longer half-life. However, ^68^Ga offers radiotracer on-site on-demand, and results in lower radiation exposure owing to its shorter half-life. As PSMA PET/CT imaging becomes more widely available and utilised in the diagnostic and therapeutic setting, it will be important to optimise imaging quality, accuracy, and cost to improve patient outcomes.

This systematic review and meta-analysis aim to compare ^68^Ga and ^18^F-based PSMA radiotracers, with a focus on comparing [^68^Ga]Ga-PSMA-11 with [^18^F]PSMA-1007 and [^18^F]DCFPyL on their diagnostic performance and normal organ distributions. The meta-analysis aims to compare the lesion uptake and benign bone uptake of the radiotracers. A brief summary of the new ^18^F PSMA radiotracers was provided. The tracers are further contrasted in the context of their production processes and costs.

## Materials/subjects and methods

This review was registered on Prospero in September 2022 (registration ID: CRD42022358864). The review was performed in a systematic approach through online searches of four scientific literature databases (MEDLINE, EMBASE, PubMed and Web of Science). The search was done in May 2023”. The search strategy is presented in a table (Table 1). Studies providing comparative analysis on the diagnostic performance of Fluorine versus Gallium PSMA PET/CT were included for analysis. The indications of the PSMA imaging included primary staging, restaging or metastatic follow-ups. All types of ^18^F-based and ^68^Ga-based PSMA radiotracers were considered. Several ^18^F based radiotracers, including[^18^F]PSMA-1007, [^18^F]DCFPyL, [^18^F]JK-PSMA-7, [^18^F]rhPSMA-7, [^18^F]AlF-PSMA-11 were compared with one ^68^Ga based tracer, [^68^Ga]Ga-PSMA-11. Study designs considered for inclusion included randomised clinical trials (RCT), prospective studies and retrospective case-control series. Case reports, conference proceedings, editorial comments, letters to the editor and review papers were excluded. Only studies published in the last 10 years in the English language were included. The search and selection of studies was performed by two independent evaluators (S.H. and S.O.) and any discrepancies were resolved by a third evaluator (N.L.).

**Table 1:** Search strategies.

1	(prostat* canc*) OR (prostat* malig*) OR (prostat* neop*)
2	(Prostate-specific membrane antigen) OR PSMA
3	Gallium OR 68Ga OR (68Ga-PSMA-11) OR (68Ga-PMSA I&T) OR (68Ga-PMSA-617)
4	Fluorine OR 18F OR DCFPyL OR (18F-PSMA-1007)
	1 AND 2 AND 3 AND 4

Studies were assessed for quality using the Quality Assessment of Diagnostic Accuracy Studies-2 (QUADAS-2). The risk of bias in patient selection was generally acceptable but high in a few studies that are retrospective head-to-head analysis as non-consecutive patients with specific clinical indications that required scans with two types of tracers were selected. Furthermore, several studies were matched pair comparisons, raising concerns regarding applicability. The risk of bias for the index test was high in some studies when no blinding of the tracer type was reported. Moreover, there is high risk of application concerns in studies that did not encourage voiding before the imaging. In terms of the reference standard, the risk of bias was high in studies that did not verify all suspicious lesions with histology. The risk of bias for timing and flow was high in several studies that did not follow up the suspicious lesions with sufficient time. Summary findings for the QUADAS-2 appraisal are illustrated in the supplementary material (Supplementary Fig. 1) [10]. The quality of each article was assessed by two independent evaluators (S.H. and S.O.) and any discrepancies were resolved by a third evaluator (N.L.).

The basic information extracted from the studies included the study nature and design, year of the study, country where the study was conducted, the indication for the scan, the sample size, imaging protocols including the time to acquisition and the injection dose. Patient characteristics were extracted when available, including the patient age, pre-scan PSA (Table 2). Quantitative analysis was considered if there are at least three studies reporting on the same outcome. The quantitative data included in the meta-analysis were lesion SUVmax and benign bone SUVmax of [^18^F]PSMA-1007 in comparison to [^68^Ga]Ga-PSMA-11; the lesion SUVmax of [^18^F]DCFPyL and [^68^Ga]Ga-PSMA-11. Additional data was requested for the meta-analysis and kindly provided by the authors of three studies [11–13]. The detection rate, sensitivity and specificity and the biodistribution of each radiotracer were compared in qualitative summaries due to the variation in studies in providing histology confirmation or clinical verification of the lesions. Most studies defined detection rate as ‘PSMA avid lesions’ rather than ‘the ratio between the number of cases correctly detected and the actual number of cases’.

**Table 2:** Characteristics of the studies included.

[^18^F]PSMA-1007										
Citation	Country	Study nature & design	Primary vs BCR	Sample size ^18^F vs ^68^Ga	Time to acquisition (min) ^18^F vs ^68^Ga	Injection dose (MBq) ^18^F vs ^68^Ga	Pre-scan preparation	Age (years) mean (SD) ^18^F vs ^68^Ga	PSA (ng/mL) mean (SD) ^18^F vs ^68^Ga	Grade group ^18^F vs ^68^Ga
Kuten [15]	Israel	Prospective head-to-head analysis	Primary	16	60 vs 45–80	NA	Hydration and voiding	56–74^a^	6.35 (3.5–19)^a^	- GG 1: 2 - GG 2: 5 - GG 3: 6 - GG 4: 3 - GG 5: 0
Draulans [11]	Belgium	Prospective matched pair analysis	Primary	10 vs 9	60	NA	NA	NA	6.3 (2.1–27.5)^a^ vs 10.1 (3.8–24.6)^a^	- GG 1: 0 - GG 2: 1 vs 3 - GG 3: 4 vs 2 - GG 4: 1 vs 2 - GG 5: 4 vs 2
Chandekar [16]	India	Prospective head-to-head analysis	Primary	40	45–60 vs 60–80	1–2 vs 3–4 MBq/kg body weight	NA	68 (8.6)	50.2 (41.6)	- GG 1: 9 - GG 2: 3 - GG 3: 11 - GG 4: 9 - GG 5: 8
Sharma [13]	India	Prospective matched pair comparison	Primary	4 vs 4	0–120	185–370 vs 37–111	NA	55.25 (4.57) vs 75.75 (10.05)	NA	NA
Zhang [38]	China	Retrospective matched pair analysis	Primary	57 vs 12	60	2.96–3.7 MBq/kg	Voiding	71 (67–75)^b^ vs 72 (68 –80)^b^	15.00 (7.30–30.58)^b^ vs 17.05 (12.47–22.65)^b^	- GG 1: 11 vs 2 - GG 2 & 3: 20 vs 6 - GG 4: 18 vs 4 - GG 5: 8 vs 0
Lengana [30]	South Africa	Prospective head-to-head analysis	BCR	21	120 vs 60	3.7 (1.24–8.25) vs 3.6 (2.01–6.3)^a^ mCi	NA	68.57 (48–78)^a^	2.55 (3.1)	- GG 1: 8 - GG 2: 8 - GG 3: 1 - GG 4: 2 - GG 5: 2
Ende [19]	Australia	Prospective head-to-head analysis	BCR	14	109 (25) vs 71 (18)	3.5 vs 2 MBq/kg	Furosemide administration in 12/14 patients	61.8 (7.1)	0.21 (0.15)	- GG 1–3: 7 - GG 4–5: 7
Rauscher [20]	Germany	Retrospective matched pair analysis	BCR	102 vs 102	94 (22) vs 54 (7)	325 (40) vs 147 (27)	NA	71 (51–84)^a^	0.87 (0.20–13.59)^a^ vs 0.91 (0.18–30.00)^a^	- GG 1–3: 63 vs 63 - GG 4–5: 39 vs 39
Alberts [18]	Switzerland	Retrospective matched pair analysis	BCR	122 vs 122	120 vs 90	243 (133–322) vs 246 (207–283)	NA	72 (54–87)^a^ vs 71 (52–85)^a^	2.23 (0.12–518)^a^ vs 2.75 (0.2–4513)^a^	7 (5 - 10)^a^
Hoffmann [17]	Germany	Retrospective matched pair analysis	BCR	128 vs 136	90 (10) vs 60 (10)	NA	NA	69.3 (8.8) vs 69.2 (8.3)	1.6 (0.1–167.1)^a^ vs 3.2 (0.1–170)^a^	- GG 1: 9 vs 6 - GG 2–3: 63 vs 82 - GG 4: 20 vs 27 - GG 5: 36 vs 21
Seifert [32]	Germany	Retrospective matched pair and head-to-head analysis	BCR	383 vs 409 matched-pair, 17 head-to-head analysis	111 (20) vs 67 (14)	350.6 (61.8) vs 133.3 (81.2)	NA	head-to-head group: 71 (69.5–74)^b^	Head-to-head group: 0.5 (0.2–1)^b^	head to head analysis: - GG 1: 1 - GG 2: 2 - GG 3: 5 - GG 4: 2 - GG 5: 1 - unknown: 6
Dietlein [26]	Germany	Retrospective head-to-head analysis	BCR	16	120 for **[**^**18**^**F]PSMA-1007**	343 (54) vs 159 (31)	NA	67.2 (7.8)	3.159	- GG 1: 1 - GG 2: 3 - GG 3: 7 - GG 4: 2 - GG 5: 2 - unknown: 1
Pattison [12]	Australia	Prospective head-to-head analysis	Primary & BCR	50 (17 primary, 33 BCR)	120–180 vs 45–60	250 vs 100–150	Hydration and voiding in both scans; fasting in ^18^F scans alone	71.8 (6.7)	2.7 (0.7–12.0)^a^	- GG 1: 1 - GG 2: 8 - GG 3: 12 - GG 4: 10 - GG 5: 11
Hoberück [21]	Germany	Retrospective head-to-head analysis	Primary & BCR	46 (10 primary, 30 BCR)	104 (11) vs 110 (18)	154 (123–175) vs 149 (111–161)	NA	71 (6.9)	3.76 (0.32–113.7)^a^	- GG 1: 2 - GG 2–3: 20 - GG 4: 6 - GG 5: 18
Dias [22]	Denmark	Retrospective matched pair analysis	Primary & BCR	10 vs 10	60–70	2 MBq/kg body weight	NA	53–78	0.2–707.7	- GG 1: 0 vs 1 - GG 2–3: 6 vs 4 - GG 4: 2 vs 3 - GG 5: 1 vs 1 - unknown: 1 vs 1

[^18^F]DCFPyL										
Citation	Country	Study nature & design	Primary vs BCR	Sample size ^18^F vs ^68^Ga	Time to acquisition (min) ^18^F vs ^68^Ga	Injection dose (MBq) ^18^F vs ^68^Ga	Pre-scan preparation	Age (years) mean (SD) ^18^F vs ^68^Ga	PSA (ng/mL) mean (SD) ^18^F vs ^68^Ga	Gleason score ^18^F vs ^68^Ga
Dietlein [23]	Switzerland	Retrospective head-to-head analysis	BCR	14	120 vs 60	318.4 (59.0) vs 128.3 (35.9)	Fasting	51–86	2.04 (0.17–50)^a^	NA
Dietlein [24]	Germany	Retrospective matched pair and head-to-head analysis	BCR	62 vs 129 matched-pair, 25 head-to-head analysis	120 vs 60	269.8 (81.8) vs 158.9 (45.1)	Fasting	RPx group: 68.4 (7) vs 70.1 (7.9), RTx group: 71.8 (8.5) vs 72.1 (6.7)	RPx group: 2.7 (3.8) vs 2.5 (2.2), RTx group: 4.1(7.5) vs 8.5 (11.1)	RPx group: - GG 1: 4 vs 13 - GG 2–3: 18 vs 33 - GG 4–5: 15 vs 21 RTx group: - GG 1: 0 vs 29 - GG 2–3: 17 vs 23 - GG 4–5: 7 vs 25
Hammes [39]	Switzerland	Retrospective head-to-head analysis	BCR	21	125 (12) vs 73 (14)	311 (61) vs 162 (54)	NA	66.5 (8.5)	NA	NA
Ferreira [25]	Australia	Retrospective head-to-head analysis	BCR	34	91 (81.25–123) vs 57 (47–68.75)^b^	3.6 (0.18) vs 1.6 (0.41) MBq/Kg	NA	67.5 (9.75)	2.0 (3.55) vs 1.9 (4.44)	NA
Jansen [40]	The Netherlands	Retrospective matched pair analysis	Primary & BCR	50 vs 87	120 (117–123) vs 65 (57–74)^b^	311.2 (301.6 –318.8) vs 139.6 (120.2 –156.5)^b^	NA	71 (66–76)^b^ vs 70 (65 –75)^b^	7.2 (2.8–17.6)^a^ vs 4.7 (1.0 –16.0)^a^	NA
Bodar [41]	The Netherlands	Prospective matched pair analysis	Primary	129 vs 189	118 (90–123)^b^ vs 48 (44–53)^b^	305.4 (240.2–318.2)^b^ vs 98.7 (92.4–104.5)^b^	NA	68.5 (62.4–72.5)^b^	10.4 (7.2–19.8)^b^	- GG 1: 3 vs 16 - GG 2: 32 vs 31 - GG 3: 39 vs 41 - GG 4: 35 vs 67 - GG 5: 20 vs 34

[^18^F]JK-PSMA-7										
Citation	Country	Study nature & design	Primary vs BCR	Sample size ^18^F vs ^68^Ga	Time to acquisition (min) ^18^F vs ^68^Ga	Injection dose (MBq) ^18^F vs ^68^Ga	Pre-scan preparation	Age (years) mean (SD) ^18^F vs ^68^Ga	PSA (ng/mL) mean (SD) ^18^F vs ^68^Ga	Gleason score ^18^F vs ^68^Ga
Dietlein [26]	Germany	Retrospective head-to-head analysis	BCR	10	120 vs 60	358 (15) vs 141 (30)	Fasting	52–76^a^	0.46–14.9	- GG 1: 1 - GG 2: 3 - GG 3: 5 - GG 4: 1 - GG 5: 0

[^18^F]rhPSMA-7										
Citation	Country	Study nature & design	Primary vs BCR	Sample size ^18^F vs ^68^Ga	Time to acquisition (min) ^18^F vs ^68^Ga	Injection dose (MBq) ^18^F vs ^68^Ga	Pre-scan preparation	Age (years) mean (SD) ^18^F vs ^68^Ga	PSA (ng/mL) mean (SD) ^18^F vs ^68^Ga	Gleason score ^18^F vs ^68^Ga
Kroenke [28]	Germany	Retrospective matched pair analysis	Primary & BCR	33 vs 33 primary, 127 vs 127 BCR	80 (20) vs 55 (9)	329 (48) vs 143 (31)	Furosemide administration	72 (52–84)^a^	Primary staging: 14 (1.37–81.00)^a^ vs 10.35 (3.80–81.56)^a^, Restaging after BCR: 0.87 (0.20–13.59)^a^ vs 2.05 (0.20–30.00)^a^	Primary staging: - GG 1–3: 10 vs 10 - GG 4–5: 23 vs 23 Restaging after BCR: - GG 1–3: 80 vs 79 - GG 4–5: 47 vs 48
[^18^F]AlF-PSMA-11										
Citation	Country	Study nature & design	Primary vs BCR	Sample size ^18^F vs ^68^Ga	Time to acquisition (min) ^18^F vs ^68^Ga	Injection dose (MBq) ^18^F vs ^68^Ga	Pre-scan preparation	Age (years) mean (SD) ^18^F vs ^68^Ga	PSA (ng/mL) mean (SD) ^18^F vs ^68^Ga	Gleason score ^18^F vs ^68^Ga
De man [27]	Belgium	Phase 3 randomised clinical trial	Primary & BCR	85 (19 primary, 66 BCR)	60 (5)	2.0 (0.2) MBq/kg	Fasting; furosemide administration	73 (67–76)^b^	Primary staging: 14.3 (7.2–27)^b^, Restaging after BCR: 0.65 (0.43–1.8)^b^	Primary staging: - GG 1–3: 5 - GG 4–5: 14 Restaging after BCR: - GG 1–3: 39 - GG 4–5: 27

*ADT* androgen deprivation therapy, *BCR* biochemical recurrence, *GG* Grade group, *Primary* primary staging, *PSA* prostate-specific antigen, *RPx* radical proctectomy, *RTx* radiotherapy^.^

Most values were given as mean (SD).

^a^Median (range).

^b^Median (IQR).

**Fig. 1:**
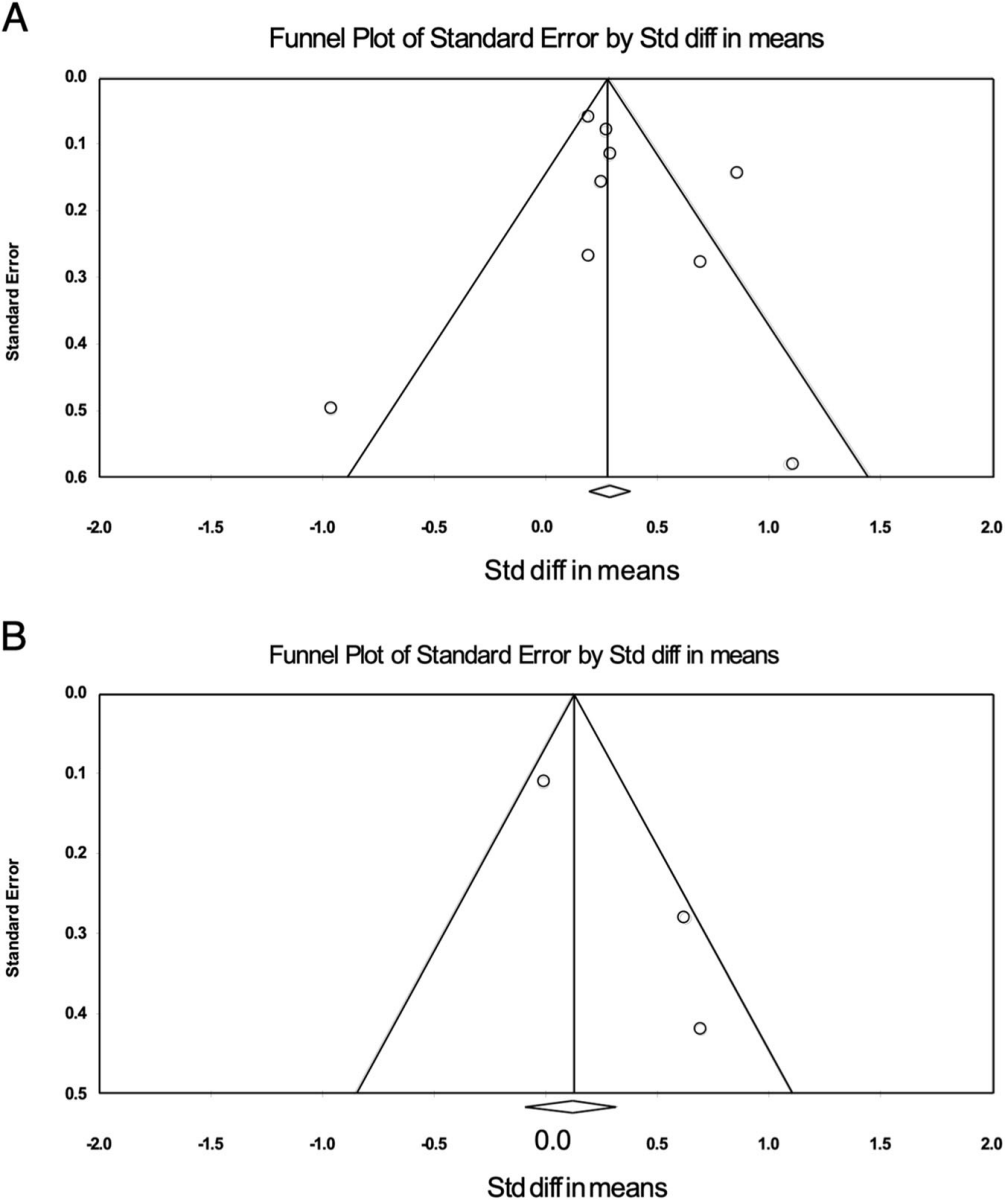
Publication bias. Funnel plots assessing publication bias in (**A**) [^18^F]PSMA-1007 and (**B**) [^18^F]DCFPyL based studies.

Extracted data were collated in Excel 2023 (Microsoft Corporation, Redmond, CA, USA) and analysis was performed using Comprehensive Meta Analysis (CMA) v.3.3.070 (Biostat Inc. Englewood, USA). The mean difference and 95% Confidence Interval were computed using paired-sample *t* test for studies that conducted intra-individual comparisons between [^18^F]PSMA-1007 or [^18^F]DCFPyL and [^68^Ga]Ga-PSMA-11; independent sample *t* test was used for studies that compared [^18^F]PSMA-1007 or [^18^F]DCFPyL and [^68^Ga]Ga-PSMA-11 in different patient cohorts with matched characteristics. Raw data were converted to means and standard deviations using SPSS v.29.0.1.0 for Mac (IBM, Armonk, NY, USA). When only median (range/interquartile range) was available, the means and standard deviations were derived using the methodology described by Wan et al. [14] and calculated with the Excel formula provided in the article. Subgroup analysis was performed for primary staging and restaging after BCR of PCa. A random-effects model was applied. *I*^2^ was used to measure the percentage of the variability in effect estimates that is due to study heterogeneity. Publication bias was assessed by funnel plots (Fig. 1). Significance was set at the 0.05 level.

## Results

**Fig. 2:**
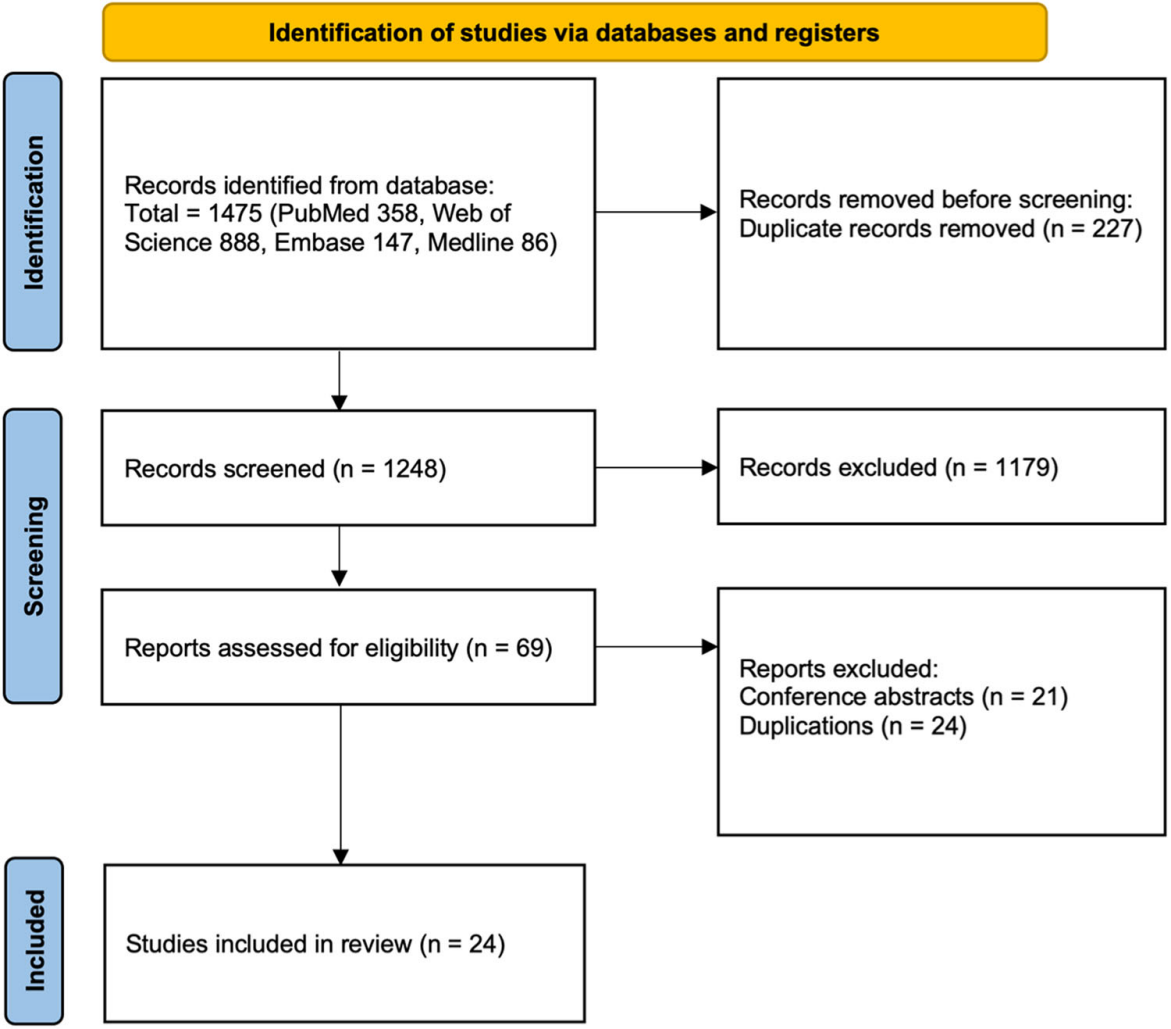
Summary of the study selection process. Studies were selected according to the Preferred Reporting Items for Systematic Review and Meta-analysis (PRISMA) guidelines. Results were summarised below.

Using the systematic search strategy, 1475 articles were identified, of which 227 were duplicate records and were excluded. Of the remaining 1248 records, 1179 were irrelevant to the research question. A further 21 were conference abstracts that could not be quality assessed and thus were excluded. From the remaining 48 articles, 24 were excluded as they contained duplicate data. This left 24 articles were suitable for assessment (Fig. 2).

### Comparison of [^18^F]PSMA-1007 vs [^68^Ga]Ga-PSMA-11

[^18^F]PSMA-1007 was evaluated against [^68^Ga]Ga-PSMA-11 by 15 studies. There were five studies on primary staging of PCa—two prospective intra-individual comparisons, two prospective matched pair analyses, one retrospective matched pair analyses. There were seven studies comparing the tracers on restaging of PCa after BCR—two prospective head-to-head analyses, one retrospective head-to-head analysis, three retrospective matched pair analyses and one retrospective matched pair and head-to-head analyses. There were three studies included patients for both primary staging of PCa and restaging after BCR—one prospective head-to-head analysis, one retrospective head-to-head analysis and one retrospective matched pair analysis.

### Detection rate of [^18^F]PSMA-1007 vs [^68^Ga]Ga-PSMA-11

Overall, [^18^F]PSMA-1007 and [^68^Ga]Ga-PSMA-11 had a high concordance in both primary staging and restaging of PCa after BCR.

### Primary staging

In primary staging of PCa, [^18^F]PSMA-1007 was found to have a higher detection rate for local lesions but without significant clinical impact. In one prospective head-to-head analysis, the sensitivity of [^18^F]PSMA-1007 vs [^68^Ga]Ga-PSMA-11 was 100% vs 85.7%; the specificity was 90.9% vs 98.2%; the positive predictive value (PPV) was 87.5% vs 96.8%; the NPV was 100% vs 91.5%, the accuracy was 94.5% vs 93.3% [15]. In another prospective head-to-head comparison, [^18^F]PSMA-1007 and [^68^Ga]Ga-PSMA-11 were both able to detect the dominant lesions [16]. However, [^18^F]PSMA-1007 was able to appreciate focal lesions better and detected three additional LN lesions [16].

### Restaging after BCR

Similarly, in restaging following BCR, [^18^F]PSMA-1007 demonstrated a comparative detection rate to [^68^Ga]Ga-PSMA-11 and was shown to have a higher detection rate for local lesions in some studies. In a matched pair comparison study that stratified study groups based on PSA, the detection rates for local lesions were found to be consistently greater in the [^18^F]PSMA-1007 group vs [^68^Ga]Ga-PSMA-11–52.9% vs 37.5% (PSA 0.2–0.5 ng/mL); 47.0% vs 46.6% (PSA 0.5–1.0 ng/mL); 52.9% vs 46.1% (PSA 1–2 ng/mL); 53.8% vs 44.4% (PSA 2–5 ng/mL); 28.5% vs 18.5% (PSA $\ge \,$ ng/mL) [17]. However, in detecting LN or distant metastasis, [^18^F]PSMA-1007 did not demonstrate a higher detection rate at all PSA levels [17]. In a study that included patients for primary staging of PCa and restaging after BCR, [^18^F]PSMA-1007 was able to detect three additional bladder wall invasions; two additional LN lesions adjacent to the ureter as [^18^F]PSMA-1007 is not associated with the retention of the tracer in the urinary tract [12]. In a prospective head-to-head analysis, the sensitivity of [^18^F]PSMA-1007 vs [^68^Ga]Ga-PSMA-11 was 88.9% vs 44.4%; the specificity was 100% vs 83.3%; PPV was 100% vs 66.7%; negative predictive value (NPV) was 92.3% vs 66.7%; accuracy was 95.5% vs 80.8%. The detection rate of [^18^F]PSMA-1007 vs [^68^Ga]Ga-PSMA-11 was 91.8% vs 86.9% (*p* = 0.68) in one matched pair comparison [18]. However, in a study that administered patients with diuretics before the scan, [^68^Ga]Ga-PSMA-11 was found to have detected three additional local lesions [19]. In addition, one matched pair comparison showed that [^18^F]PSMA-1007 (26.5%) has a lower detection rate for local lesions in comparison with [^68^Ga]Ga-PSMA-11 (32.4%) [20]. In another head-to-head comparison that include both patients for primary staging and restaging after BCR, both [^18^F]PSMA-1007 and [^68^Ga]Ga-PSMA-11 detected two additional local lesions [21].

### Lesion SUVmax of [^18^F]PSMA-1007 vs [^68^Ga]Ga-PSMA-11

**Fig. 3:**
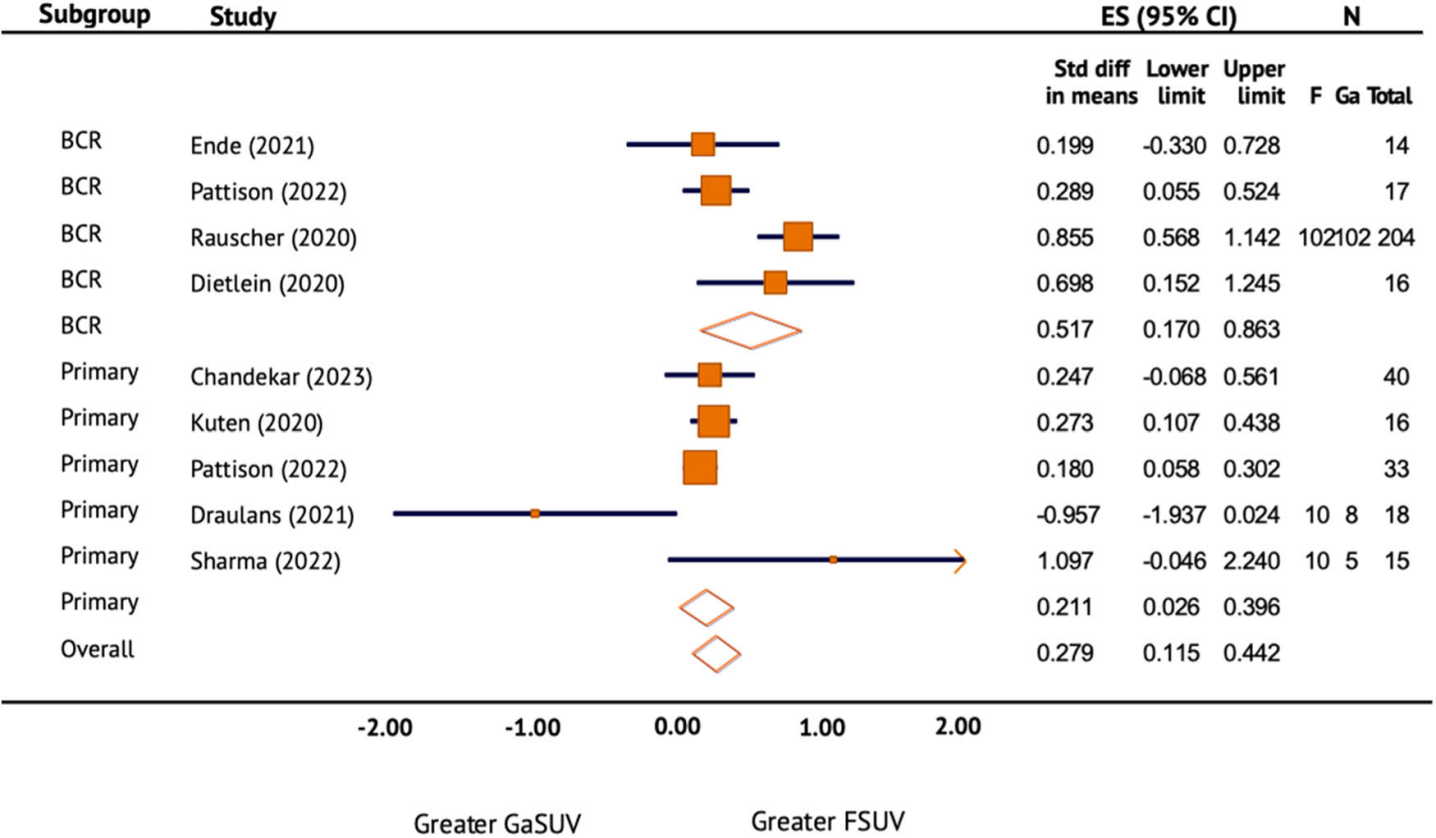
Lesion SUVmax of [18F]PSMA-1007 vs [68Ga]Ga-PSMA-11. Forest plot of the standard difference in means and 95% confidence interval of lesion SUVmax of [^18^F]PSMA-1007 in comparison with [^68^Ga]Ga-PSMA-11 on prostate-specific membrane antigen positron emission tomography (PSMA PET/CT) by primary staging and restaging after biochemical recurrence (BCR) of prostate cancer. ES effect size.

A meta-analysis was conducted to evaluate the lesion uptake of [^18^F]PSMA-1007 in comparison with [^68^Ga]Ga-PSMA-11 through eight studies (Fig. 3). Five of the studies were intra-individual comparisons. The remaining three studies were matched pair comparisons. The meta-analysis found that the lesion SUVmax of [^18^F]PSMA-1007 was significantly greater than [^68^Ga]Ga-PSMA-11. The overall effect size (ES) measured by standard difference in means was 0.279 (95% CI 0.115–0.442). In subgroup analysis, the ES was found to be greater in the restaging group (ES = 0.517, 95% CI 0.17–0.863). The effect size was 0.211 (95% CI 0.026–0.396). There was substantial heterogeneity between groups and within both subgroups (*I*^2^ > 50%).

### Benign bone SUVmax of [^18^F]PSMA-1007 vs [^68^Ga]Ga-PSMA-11

[^18^F]PSMA-1007 was observed to have a higher benign bone uptake and lead to false positives in some studies. It reported one false positive bone lesion in one study that included patients with BCR [19]; and five positive bone lesions in another study that included patients with primary PCa or BCR [12]. The Pattison study also observed a significantly greater bone SUVmean in [^18^F]PSMA-1007 in comparison to [^68^Ga]Ga-PSMA-11 (1.5 vs 0.8, *p* < 0.001) [12]. Dias et al. also found a higher background signal in the bone [22].

**Fig. 4:**
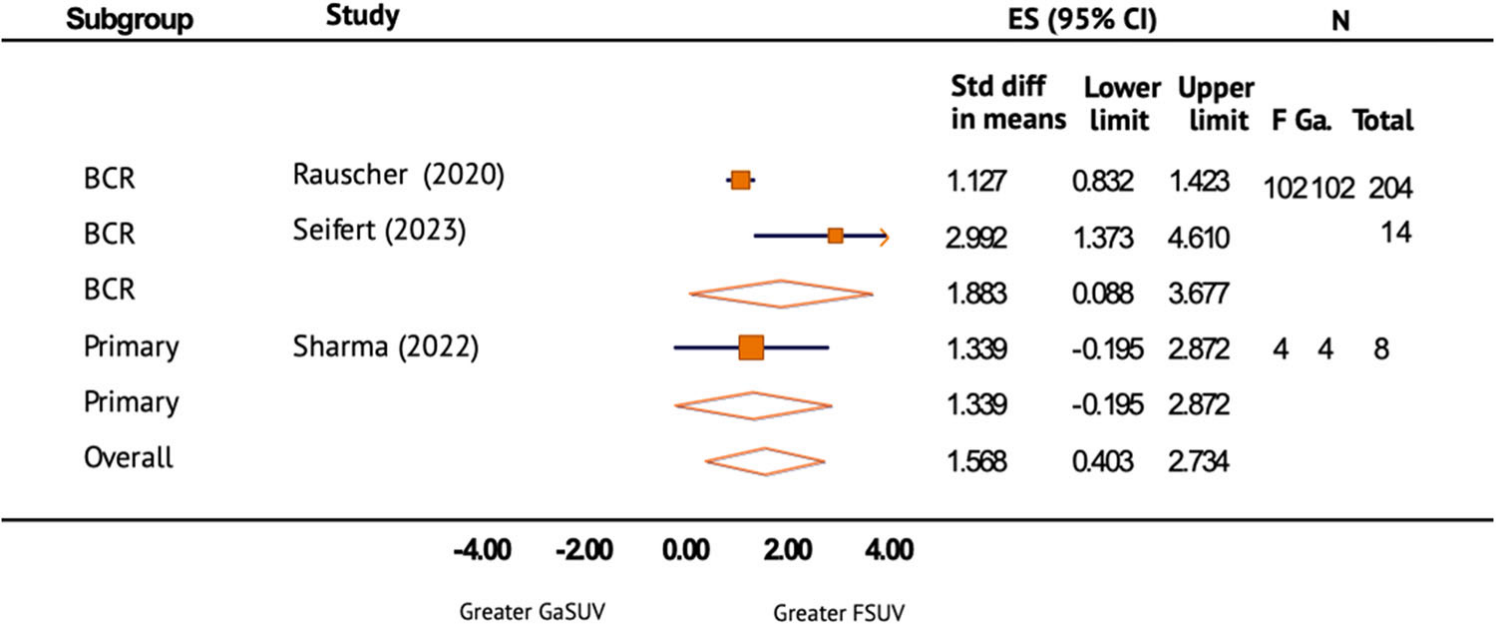
Benign bone SUVmax of [18F]PSMA-1007 vs [68Ga]Ga-PSMA-11. Forest plot of the standard difference in means and 95% confidence interval of benign bone SUVmax of [^18^F]PSMA-1007 in comparison with [^68^Ga]Ga-PSMA-11 on prostate-specific membrane antigen position emission tomography (PSMA PET/CT) by primary staging and restaging after biochemical recurrence (BCR) of prostate cancer. ES effect size.

A meta-analysis with three studies was conducted to further evaluate the benign bone uptake of [^18^F]PSMA-1007 in comparison with [^68^Ga]Ga-PSMA-11 (Fig. 4). In the meta-analysis, the benign bone SUVmax of [^18^F]PSMA-1007 was found to be significantly greater than [^68^Ga]Ga-PSMA-11. The overall effect size (ES) was 1.568 (95% CI 0.403–2.734). There was substantial heterogeneity between groups (*I*^2^ > 50%).

### Other organ distribution of [^18^F]PSMA-1007 vs [^68^Ga]Ga-PSMA-11

[^18^F]PSMA-1007 was also observed to have a higher liver uptake in comparison to [^68^Ga]Ga-PSMA-11. The mean liver SUVmean of [^18^F]PSMA-1007 and [^68^Ga]Ga-PSMA-11 was 13.0 vs 7.0, *p* < 0.001 [21]; 11.9 vs 4.4, *p* < 0.001 [12];12.17 vs 4.85 [13] in three respective studies; the mean SUVmax was 11.82 vs 5.37, *p* < 0.0001 [19]; 20.50 (2.83) vs 11.15 (4.23) [13] in two respective studies.

In contrary, significantly lower urinary bladder uptake of [^18^F]PSMA-1007 was reported in four studies and lower kidney uptake in one study. The median urinary bladder SUVmean of [^18^F]PSMA-1007 and [^68^Ga]Ga-PSMA-11 were 3.0 vs 14.8, *p* < 0.001 [12]; 3.66 vs 25.35, *p* < 0.001 [15]; 2.90 (1.14) vs 7.40 (3.55) [13] in three respective studies; mean SUVmax was 3.46 vs 9.67, *p* = 0.0042 [19]. A similar fold of difference was observed in the kidney (mean kidney SUVmean of [^18^F]PSMA-1007 and [^68^Ga]Ga-PSMA-11 were 15.18 and 25.89, respectively [13]).

### [^18^F]DCFPyL vs [^68^Ga]Ga-PSMA-11

#### Detection rate of [^18^F]DCFPyL vs [^68^Ga]Ga-PSMA-11

The detection rate of [^18^F]DCFPyL was evaluated against [^68^Ga]Ga-PSMA-11 by Dietlein and colleagues in a head-to-head analysis and a matched pair analysis [23, 24]. [^18^F]DCFPyL was consistently observed to have a greater detection rate. In the matched pair analysis [24], [^18^F]DCFPyL was observed to have a significantly higher detection rate than [^68^Ga]Ga-PSMA-11 (88% vs 65%, *p* = 0.042) when PSA is low (0.5–3.5 ng/mL). However, few lesions were verified. No clinical impacts were reported.

#### Lesion SUVmax of [^18^F]DCFPyL vs [^68^Ga]Ga-PSMA-11

**Fig. 5:**
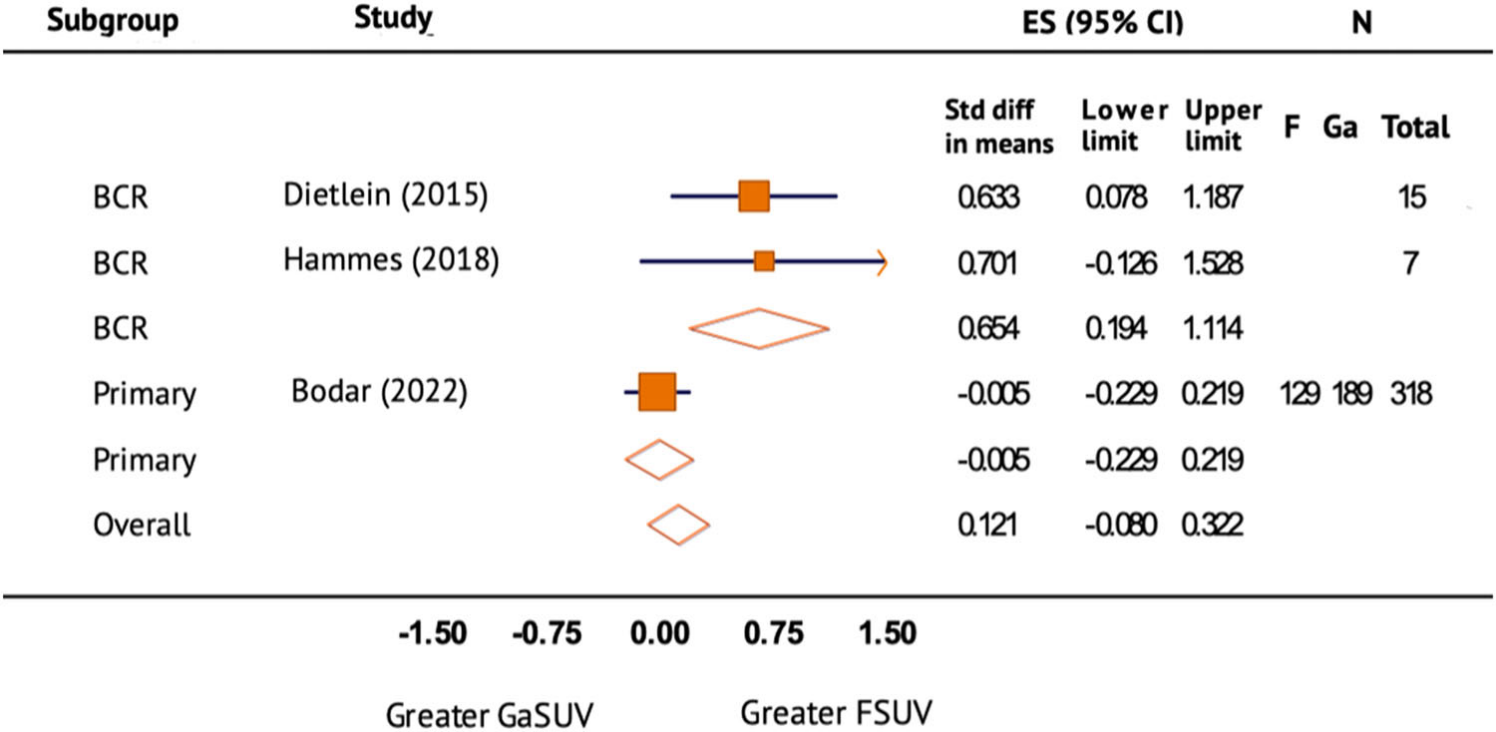
Lesion SUVmax of [18F]DCFPyL vs [68Ga]Ga-PSMA-11. Forest plot of the standard difference in means and 95% confidence interval of lesion SUVmax of [^18^F]DCFPyL in comparison with [^68^Ga]Ga-PSMA-11 on prostate-specific membrane antigen positron emission tomography (PSMA PET/CT) by primary staging and restaging after biochemical recurrence (BCR) of prostate cancer. ES effect size.

In evaluating the lesion uptake of [^18^F]DCFPyL in comparison with [^68^Ga]Ga-PSMA-11, a meta-analysis was conducted using three studies (Fig. 5). The lesion SUVmax of [^18^F]DCFPyL greater than [^68^Ga]Ga-PSMA-11 but the difference was not significant. The overall effect size (ES) was 0.121 (95% CI −0.080–0.322). There was substantial heterogeneity between groups and within both subgroups (*I*^2^ > 50%).

#### Organ distributions of [^18^F]DCFPyL vs [^68^Ga]Ga-PSMA-11

[^18^F]DCFPyL was observed to have a similar biodistribution to [^68^Ga]GaPSMA-11 except for its significantly lower kidney uptake. The mean kidney SUVmean of [^18^F]DCFPyL and [^68^Ga]Ga-PSMA-11 was 19.6 vs 31.7, *p* = 0.001 [25]; the SUVpeak was 40.0 vs 59.6, *p* < 0.001 [23]. Liver and urinary bladder uptakes were similar between [^18^F]DCFPyL and [^68^Ga]Ga-PSMA-11 [23, 25].

#### [^18^F]JK-PSMA-7

[^18^F]JK-PSMA-7 was evaluated against [^68^Ga]Ga-PSMA-11 by Dietlein et al. in one pilot study that included ten patients who had undergone a [^68^Ga]Ga-PSMA-11 scan, but the results were negative or inconclusive in five of the patients [26]. [^18^F]JK-PSMA-7 was observed to have a higher detection rate. However, only one of the additional lesions underwent verification. Nevertheless, this additional verified lesion led to subsequent radiotherapy which would not have been performed had [^68^Ga]Ga-PSMA-11 been used alone.

#### [^18^F] AlF-PSMA-11

In a phase 3 randomised clinical trial (19 primary staging, 66 BCR/PSA persistence), [^18^F]AlF-PSMA-11 was observed to have led to more upstaging of the miTNM score [27]. However, most lesions were not verified by histology. No clinical impacts were mentioned.

#### [^18^F]rhPSMA-7

[^18^F]rhPSMA-7 was evaluated against [^68^Ga]Ga-PSMA-11 in a retrospective matched pair analysis (33 primary staging, 127 restaging after BCR in each group) [28]. [^18^F]rhPSMA-7 was observed to have higher detection rates for local lesions and distant lesions but a lower detection rate for lymph node (LN) lesions in comparison to [^68^Ga]Ga-PSMA-11. The lesions were not verified. No clinical impacts were mentioned.

## Discussion

### Overall comparison between ^18^F and ^68^Ga-based PSMA radiotracers

Overall, ^18^F-based PSMA radiotracers demonstrated a higher SUVmax and a marginally higher detection rate, although these differences were not significant and there is a paucity of high-quality head-to-head comparative data. The improved image spatial resolution secondary to the lower positron energy of ^18^F versus ^68^Ga (0.65 vs 1.90 meV), which results in a shorter positron range (Rmax 2.4 mm vs 9.2 mm) might favour ^18^F [29]. In addition, the injection dose of ^18^F is higher in all studies due to its greater production yield and some studies use a longer time uptake time making direct comparison to ^68^Ga difficult. The longer half-life and higher administered activities result in higher radiation exposure to patients and staff. The clinical impact of the difference made by ^18^F was either not mentioned or was shown to be limited in most studies. Different ^18^F-based PSMA tracers have some unique features to compare with [^68^Ga]Ga-PSMA-11 and they are discussed in the below sections.

### [^18^F]PSMA-1007

The main advantage of [^18^F]PSMA-1007 observed in this review was its greater locoregional lesion detection rate and accuracy in local lesion delineation [12, 15, 17, 21, 30, 31]. This is likely secondary to its greater lesion SUV uptake and predominant hepatobiliary excretion route. In our study, the effect size of lesion SUVmax of [^18^F]PSMA-1007 in comparison to [^68^Ga]Ga-PSMA-11 was even greater in patients with BCR. This could be related to ^18^F-based tracers’ higher sensitivity when PSA is lower [24]. In comparison, the predominant urinary excretion of [^68^Ga]Ga-PSMA-11 is likely to obscure local lesions near the prostate. However, decreasing the urinary excretion of [^68^Ga]Ga-PSMA-11 through the administration of diuretics holds promise for reducing this obscuring effect, with one study observing a greater local lesion detection rate with [^68^Ga]Ga-PSMA-11 after administering diuretics prior to the scan [19]. A main pitfall of [^18^F]PSMA-1007 was its greater rate of false positive bone uptakes observed by a number of studies [12, 13, 16, 19, 20, 32]. [^18^F]PSMA-1007 was determined to be less cost effective as more effort is needed to observe morphological correlations on CT and follow-ups are required due to these benign bone uptakes [18]. However, Arnfield and colleagues proposed that the false positives could be reduced by increasing the cut-point SUVmax of [^18^F]PSMA-1007 to 7.2 in detecting bone lesions [33]. The other pitfall of [^18^F]PSMA-1007 reported by the Pattison study was its intense liver uptake, which obscured adjacent metastatic lesions [12]. A correlating CT was required to capture the lesion. Additionally, the benign ganglia uptake is also greater in [^18^F]PSMA-1007, which should be noted by inexperienced readers and not be misinterpreted as lymph note metastasis [12, 21]. The detection rate of [^18^F]PSMA-1007 was observed to be lower in one matched pair study [20]. This could be related to the lower median PSA level in the ^18^F group. Nevertheless, the study observed a considerably higher number of local recurrences directly adjacent to the urinary bladder in ^18^F, in accordance with the findings in other studies.

### [^18^F]DCFPyL

[^18^F]DCFPyL was found to have a similar biodistribution as [^68^Ga]Ga-PSMA-11 including a similar bladder uptake [25]. In keeping with this, [^18^F]DCFPyL did not demonstrate a significantly higher local lesion detection rate. [^18^F]DCFPyL was reported to have an overall higher detection rate. This could be contributed by the better image spatial resolution provided by ^18^F. However, due to the retrospective nature of the matched pair analysis [24], there could be contributed by reporting bias. As [^18^F]DCFPyL is a newer agent, reporters would have had more experience through reading [^68^Ga]Ga-PSMA-11 scans, resulting in reporting a higher detection rate with [^18^F]DCFPyL. Additionally, [^18^F]DCFPyL is not associated with increased coeliac ganglia uptake [23].

### New ^18^F-based PSMA tracers

[^18^F]JK-PSMA-7, [^18^F]rhPSMA-7 and [^18^F]AlF-PSMA-11 all demonstrated marginally greater detection rates in comparison with [^68^Ga]PSMA-11. The sensitivity of [^18^F]JK-PSMA-7 was proven by its ability to detect more lesions in small anatomic structures [26]. [^18^F]-rhPSMA-7 is likely to have a lower urinary excretion in comparison with [^68^Ga]Ga-PSMA-11 as, when diuretics were administered prior to the scan, [^18^F]rhPSMA-7 remained more effective at detecting lesions adjacent to the bladder [28]. There were two additional preclinical studies comparing [^18^F]AlF-PSMA-11 with [^68^Ga]Ga-PSMA-11. In both studies, [^18^F]AlF-PSMA-11 was observed to have limited hepatobiliary and urinary excretions. In the matched pair comparison (1 vs 3 mice in the ^18^F and ^68^Ga group). However, Kroenke et al. also observed higher ganglion uptake by [^18^F]AlF-PSMA-11 [28].

### Production and cost

The production of [^18^F]PSMA-1007 was determined to be cheaper than [^68^Ga]Ga-PSMA-11 in one study assessing the production process, maintenance and waste disposal [34]. The production of ^68^Ga is more challenging as it requires an on-site generator. The transportation of ^68^Ga from another site is difficult due to its short half-life. However, for sites that already have a ^68^Ge/^68^Ga generator, cost and access may cheaper. In addition, the current production yield of ^68^Ga is less than ^18^F, resulting in a lower injection dose, which may impact the image resolution. However, recent studies have shown that higher radiochemical yield of ^68^Ga may be enabled by a cyclotron [35]. The labelling of ^68^Ga is easier in comparison to ^18^F. Labelling of ^68^Ga is facilitated by an automated system at an ambient temperature. In contrast, most ^18^F-based PSMA tracers need to be labelled manually with a specific temperature requirement. However, automated radiosynthesis is available for certain types of ^18^F tracers, such as [^18^F]AlF-PSMA-11 though further optimisation is needed [36].

### Limitations

This review has several limitations. Due to the limited number of studies (less than ten) available for meta-analysis, there was a strong possibility of bias in *I*^2^ [37]. There were several confounding factors, including the individual variations in the matched pair comparisons, the use of different PET/CT scanners, and different pre-scan voiding status. There was a risk of bias secondary to the lack of histological verifications and lack of studies on the neo-^18^F-based tracers. The scope of the current literature is limited by the lack of data collection regarding clinical impacts. In addition, there is no study comparing Fluorine tracers with other types of Gallium tracers such as [^68^Ga]Ga-PSMA-I&T and [^68^Ga]Ga-PSMA-617. Furthermore, due to the scope of this review, the therapeutic use of PSMA radiotracers were not considered. Lastly, the individual studies on ^68^Ga and ^18^F based PSMA radiotracers were not included due to the presence of confounding factors.

## Conclusions

[^18^F]DCFPyL was assessed to be a suitable alternative to [^68^Ga]Ga-PSMA-11 in PCa diagnosis and staging due to its similar lesion uptake rate with no increase in benign uptakes. [^18^F]PSMA-1007 was assessed to be less preferrable to [^68^Ga]Ga-PSMA-11 due to its significant number of benign bone uptakes. Although [^18^F]PSMA-1007 was able to detect more locoregional lesions due to its lower urinary excretions, the use of diuretics prior to the scan facilitated [^68^Ga]Ga-PSMA-11 to achieve a similar local lesion detection rate in comparison to [^18^F]PSMA-1007. Overall, there was not enough evidence in differentiating [^18^F]DCFPyL and [^68^Ga]Ga-PSMA-11 in their clinical impacts. The decision to use [^18^F]DCFPyL or [^68^Ga]Ga-PSMA-11 is largely based on infrastructure available at the individual health service.

## Supplementary information

QUADAS-2 risk assessment
