# Supplementary material for: Comparison of 18F-based PSMA radiotracers with [68Ga]Ga-PSMA-11 in PET/CT imaging of prostate cancer—a systematic review and meta-analysis
Source: Prostate Cancer Prostatic Dis. 2023 Nov 28;27(4):654–64. doi: 10.1038/s41391-023-00755-2 (PMC11543591; doi:10.1038/s41391-023-00755-2)
Supplement: Supplementary file 1 — QUADAS-2 risk assessment [file 41391_2023_755_MOESM1_ESM.docx]

| **Study** | **RISK OF BIAS** | | | | **APPLICABILITY CONCERNS** | | |
| --- | --- | --- | --- | --- | --- | --- | --- |
|  | **PATIENT SELECTION** | **INDEX TEST** | **REFERENCE STANDARD** | **FLOW AND TIMING** | **PATIENT SELECTION** | **INDEX TEST** | **REFERENCE STANDARD** |
| Alberts (2021) [1] | ☺ | ☺ | ☺ | ☹ | ☹ | ☹ | ☹ |
| Bodar (2022) [2] | ☺ | ☺ | ☺ | ☺ | ☹ | ☹ | ☹ |
| Chandekar (2023) [3] | ☺ | ☺ | ☺ | ☹ | ☺ | ☹ | ☺ |
| De man (2022) [4] | ☺ | ☺ | ☺ | ☹ | ☺ | ☺ | ☺ |
| Dias (2023) [5] | ☺ | ☺ | ☺ | ☺ | ☹ | ☹ | ☹ |
| Dietlein (2015) [6] | ☹ | ☹ | ☹ | ☹ | ☺ | ☹ | ☺ |
| Dietlein (2017) [7] | ☺ | ☺ | ☺ | ☹ | ☹ | ☹ | ☹ |
| Dietlein (2020) [8] | ☹ | ☹ | ☹ | ☹ | ☺ | ☹ | ☺ |
| Dietlein (2020) [9] | ☹ | ☹ | ☹ | ☹ | ☺ | ☹ | ☺ |
| Draulans (2021) [10] | ☺ | ☺ | ☺ | ☺ | ☹ | ☹ | ☹ |
| Ende (2021) [11] | ☺ | ☺ | ☺ | ☺ | ☺ | ☺ | ☺ |
| Ferreira (2019) [12] | ☹ | ☺ | ☺ | ☹ | ☺ | ☹ | ☺ |
| Hammes (2018) [13] | ☹ | ☹ | ☹ | ☹ | ☺ | ☹ | ☺ |
| Hoberück (2021) [14] | ☹ | ☹ | ☹ | ☹ | ☺ | ☹ | ☺ |
| Hoffmann (2022) [15] | ☺ | ☺ | ☺ | ☹ | ☹ | ☹ | ☹ |
| Jansen (2019) [16] | ☺ | ☺ | ☺ | ☺ | ☹ | ☹ | ☹ |
| Kroenke (2021) [17] | ☺ | ☺ | ☺ | ☹ | ☹ | ☹ | ☹ |
| Kuten (2020) [18] | ☺ | ☺ | ☺ | ☺ | ☺ | ☺ | ☺ |
| Lengana (2021) [19] | ☺ | ☺ | ☺ | ☺ | ☺ | ☹ | ☺ |
| Pattison (2022) [20] | ☺ | ☺ | ☺ | ☹ | ☺ | ☺ | ☺ |
| Rauscher (2020) [21] | ☺ | ☺ | ☺ | ☹ | ☹ | ☹ | ☹ |
| Seifert (2023) [22] | ☺ | ☺ | ☺ | ☺ | ☹ | ☹ | ☹ |
| Sharma (2022) [23] | ☹ | ☺ | ☺ | ☺ | ☹ | ☹ | ☹ |
| Zhang (2022) [24] | ☺ | ☺ | ☺ | ☺ | ☹ | ☹ | ☹ |

☺Low Risk ☹High Risk ? Unclear Risk


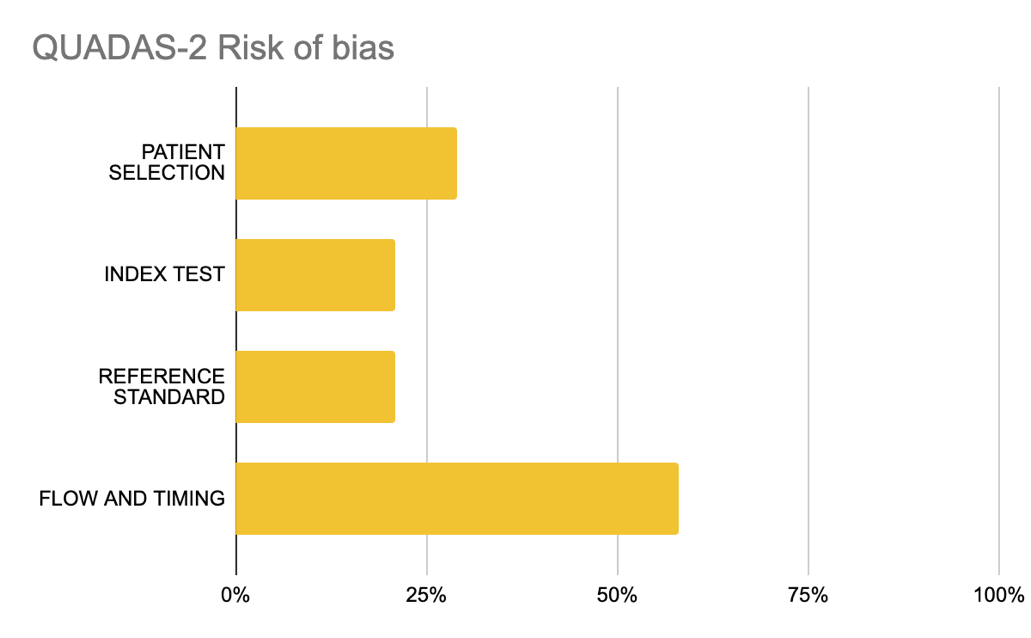


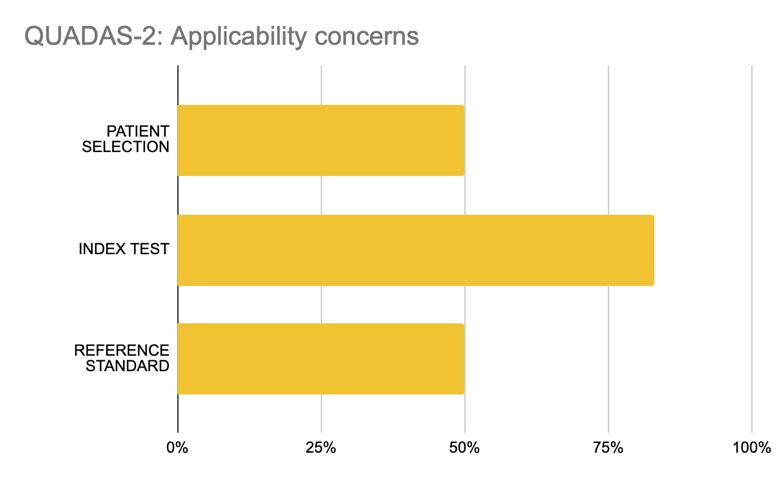


Supplementary Figure 1 – (A) Appraisal of the quality of the studies included according to the Quality Assessment for Diagnostic Studies-2 (QUADAS-2) tool [25]. (B) Summary of QUADAS-2 risk of bias. (C) Summary of QUADAS-2 applicability concerns.

Reference

1. Alberts I, Mingels C, Zacho HD, Lanz S, Schoder H, Rominger A, et al. Comparing the clinical performance and cost efficacy of [(68)Ga]Ga-PSMA-11 and [(18)F]PSMA-1007 in the diagnosis of recurrent prostate cancer: a Markov chain decision analysis. Eur J Nucl Med Mol Imaging. 2021. doi:10.1007/s00259-021-05620-9.

2. Bodar YJL, Veerman H, Meijer D, de Bie K, van Leeuwen PJ, Donswijk ML, et al. Standardised uptake values as determined on prostate‐specific membrane antigen positron emission tomography/computed tomography is associated with oncological outcomes in patients with prostate cancer. BJU International. 2022;129:768-76. doi:10.1111/bju.15710.

3. Chandekar KR, Singh H, Kumar R, Kumar S, Kakkar N, Mittal BR, et al. Comparison of 18 F-PSMA-1007 PET/CT With 68 Ga-PSMA-11 PET/CT for Initial Staging in Intermediate- and High-Risk Prostate Cancer. Clin Nucl Med. 2023;48:e1-e8. doi:10.1097/RLU.0000000000004430.

4. De Man K, Van Laeken N, Schelfhout V, Fendler WP, Lambert B, Kersemans K, et al. (18)F-PSMA-11 Versus (68)Ga-PSMA-11 Positron Emission Tomography/Computed Tomography for Staging and Biochemical Recurrence of Prostate Cancer: A Prospective Double-blind Randomised Cross-over Trial. Eur Urol. 2022. doi:10.1016/j.eururo.2022.05.010.

5. Dias AH, Jochumsen MR, Zacho HD, Munk OL, Gormsen LC. Multiparametric dynamic whole-body PSMA PET/CT using [(68)Ga]Ga-PSMA-11 and [(18)F]PSMA-1007. EJNMMI Res. 2023;13:31. doi:10.1186/s13550-023-00981-8.

6. Dietlein M, Kobe C, Kuhnert G, Stockter S, Fischer T, Schomacker K, et al. Comparison of [(18)F]DCFPyL and [ (68)Ga]Ga-PSMA-HBED-CC for PSMA-PET Imaging in Patients with Relapsed Prostate Cancer. Mol Imaging Biol. 2015;17:575-84. doi:10.1007/s11307-015-0866-0.

7. Dietlein F, Kobe C, Neubauer S, Schmidt M, Stockter S, Fischer T, et al. PSA-Stratified Performance of (18)F- and (68)Ga-PSMA PET in Patients with Biochemical Recurrence of Prostate Cancer. J Nucl Med. 2017;58:947-52. doi:10.2967/jnumed.116.185538.

8. Dietlein F, Hohberg M, Kobe C, Zlatopolskiy BD, Krapf P, Endepols H, et al. An (18)F-Labeled PSMA Ligand for PET/CT of Prostate Cancer: First-in-Humans Observational Study and Clinical Experience with (18)F-JK-PSMA-7 During the First Year of Application. J Nucl Med. 2020;61:202-9. doi:10.2967/jnumed.119.229542.

9. Dietlein F, Kobe C, Hohberg M, Zlatopolskiy BD, Krapf P, Endepols H, et al. Intraindividual Comparison of (18)F-PSMA-1007 with Renally Excreted PSMA Ligands for PSMA PET Imaging in Patients with Relapsed Prostate Cancer. J Nucl Med. 2020;61:729-34. doi:10.2967/jnumed.119.234898.

10. Draulans C, Pos F, Smeenk RJ, Kerkmeijer L, Vogel WV, Nagarajah J, et al. (68)Ga-PSMA-11 PET, (18)F-PSMA-1007 PET, and MRI for Gross Tumor Volume Delineation in Primary Prostate Cancer: Intermodality and Intertracer Variability. Pract Radiat Oncol. 2021;11:202-11. doi:10.1016/j.prro.2020.11.006.

11. Emmett LE, J.; Amin, A.; Sheehan-Dare, G.; Cusick, T. . Pilot Trial Comparing the Performance of 68Ga-PSMA-11 PET/CT to 18F-PSMA-1007 PET/CT in the Detection of Prostate Cancer Recurrence in Men

with Rising PSA Following Radical Prostatectomy. J Radiol Med Imaging. 2021;4:1039.

12. Ferreira G, Iravani A, Hofman MS, Hicks RJ. Intra-individual comparison of (68)Ga-PSMA-11 and (18)F-DCFPyL normal-organ biodistribution. Cancer Imaging. 2019;19:23. doi:10.1186/s40644-019-0211-y.

13. Hammes J, Hohberg M, Tager P, Wild M, Zlatopolskiy B, Krapf P, et al. Uptake in non-affected bone tissue does not differ between [18F]-DCFPyL and [68Ga]-HBED-CC PSMA PET/CT. PLoS One. 2018;13:e0209613. doi:10.1371/journal.pone.0209613.

14. Hoberuck S, Lock S, Borkowetz A, Sommer U, Winzer R, Zophel K, et al. Intraindividual comparison of [(68) Ga]-Ga-PSMA-11 and [(18)F]-F-PSMA-1007 in prostate cancer patients: a retrospective single-center analysis. EJNMMI Res. 2021;11:109. doi:10.1186/s13550-021-00845-z.

15. Hoffmann MA, von Eyben FE, Fischer N, Rosar F, Muller-Hubenthal J, Buchholz HG, et al. Comparison of [(18)F]PSMA-1007 with [(68)Ga]Ga-PSMA-11 PET/CT in Restaging of Prostate Cancer Patients with PSA Relapse. Cancers (Basel). 2022;14. doi:10.3390/cancers14061479.

16. Jansen BHE, Kramer GM, Cysouw MCF, Yaqub MM, de Keizer B, Lavalaye J, et al. Healthy Tissue Uptake of (68)Ga-Prostate-Specific Membrane Antigen, (18)F-DCFPyL, (18)F-Fluoromethylcholine, and (18)F-Dihydrotestosterone. J Nucl Med. 2019;60:1111-7. doi:10.2967/jnumed.118.222505.

17. Kroenke M, Mirzoyan L, Horn T, Peeken JC, Wurzer A, Wester HJ, et al. Matched-Pair Comparison of (68)Ga-PSMA-11 and (18)F-rhPSMA-7 PET/CT in Patients with Primary and Biochemical Recurrence of Prostate Cancer: Frequency of Non-Tumor-Related Uptake and Tumor Positivity. J Nucl Med. 2021;62:1082-8. doi:10.2967/jnumed.120.251447.

18. Kuten J, Fahoum I, Savin Z, Shamni O, Gitstein G, Hershkovitz D, et al. Head-to-Head Comparison of (68)Ga-PSMA-11 with (18)F-PSMA-1007 PET/CT in Staging Prostate Cancer Using Histopathology and Immunohistochemical Analysis as a Reference Standard. J Nucl Med. 2020;61:527-32. doi:10.2967/jnumed.119.234187.

19. Lengana T, Lawal IO, Rensburg CV, Mokoala KMG, Moshokoa E, Ridgard T, et al. A comparison of the diagnostic performance of (18)F-PSMA-1007 and (68)GA-PSMA-11 in the same patients presenting with early biochemical recurrence. Hell J Nucl Med. 2021;24:178-85. doi:10.1967/s002449912401.

20. Pattison DA, Debowski M, Gulhane B, Arnfield EG, Pelecanos AM, Garcia PL, et al. Prospective intra-individual blinded comparison of [(18)F]PSMA-1007 and [(68) Ga]Ga-PSMA-11 PET/CT imaging in patients with confirmed prostate cancer. Eur J Nucl Med Mol Imaging. 2022;49:763-76. doi:10.1007/s00259-021-05520-y.

21. Rauscher I, Kronke M, Konig M, Gafita A, Maurer T, Horn T, et al. Matched-Pair Comparison of (68)Ga-PSMA-11 PET/CT and (18)F-PSMA-1007 PET/CT: Frequency of Pitfalls and Detection Efficacy in Biochemical Recurrence After Radical Prostatectomy. J Nucl Med. 2020;61:51-7. doi:10.2967/jnumed.119.229187.

22. Seifert R, Telli T, Opitz M, Barbato F, Berliner C, Nader M, et al. Unspecific (18)F-PSMA-1007 Bone Uptake Evaluated Through PSMA-11 PET, Bone Scanning, and MRI Triple Validation in Patients with Biochemical Recurrence of Prostate Cancer. J Nucl Med. 2023;64:738-43. doi:10.2967/jnumed.118.215434.

23. Sharma P, Watts A, Singh H. Comparison of Internal Dosimetry of 18 F-PSMA-1007 and 68 Ga-PSMA-11-HBED-CC. Clin Nucl Med. 2022. doi:10.1097/RLU.0000000000004353.

24. Zhang YN, Lu ZG, Wang SD, Lu X, Zhu LL, Yang X, et al. Gross tumor volume delineation in primary prostate cancer on (18)F-PSMA-1007 PET/MRI and (68)Ga-PSMA-11 PET/MRI. Cancer Imaging. 2022;22:36. doi:10.1186/s40644-022-00475-1.

25. Whiting PF RA, Westwood ME, et al. QUADAS-2: a revised tool for the quality assessment of diagnostic accuracy studies. Ann Intern Med 2011;155:529–36.
